# Integrating Insults: Using Fault Tree Analysis to Guide Schizophrenia Research across Levels of Analysis

**DOI:** 10.3389/fnhum.2015.00698

**Published:** 2016-01-06

**Authors:** Angus W. MacDonald III, Jennifer L. Zick, Matthew V. Chafee, Theoden I. Netoff

**Affiliations:** ^1^Department of Psychology, Translational Research in Cognitive and Affective Mechanisms, University of MinnesotaMinneapolis, MN, USA; ^2^Department of Neuroscience, University of Minnesota School of MedicineMinneapolis, MN, USA; ^3^Veterans Affairs Medical CenterMinneapolis, MN, USA; ^4^Department of Biomedical Engineering, University of MinnesotaMinneapolis, MN, USA

**Keywords:** reliability engineering, fault tree analysis, schizophrenia, DSM-5, research domain criteria, psychosis, NMDA receptor

## Abstract

The grand challenges of schizophrenia research are linking the causes of the disorder to its symptoms and finding ways to overcome those symptoms. We argue that the field will be unable to address these challenges within psychiatry’s standard neo-Kraepelinian (DSM) perspective. At the same time the current corrective, based in molecular genetics and cognitive neuroscience, is also likely to flounder due to its neglect for psychiatry’s syndromal structure. We suggest adopting a new approach long used in reliability engineering, which also serves as a synthesis of these approaches. This approach, known as fault tree analysis, can be combined with extant neuroscientific data collection and computational modeling efforts to uncover the causal structures underlying the cognitive and affective failures in people with schizophrenia as well as other complex psychiatric phenomena. By making explicit how causes combine from basic faults to downstream failures, this approach makes affordances for: (1) causes that are neither necessary nor sufficient in and of themselves; (2) within-diagnosis heterogeneity; and (3) between diagnosis co-morbidity.

The current framework for categorizing psychiatric disorders such as schizophrenia impedes progress toward the goals of understanding the causes and cures for these illnesses. This article will describe an alternative framework derived from reliability engineering, a field developed to study the way in which complex systems break down. Changing a *framework* is more than window dressing: frameworks carry the assumptions and affordances that invisibly guide our work. We will first critique two prominent frameworks in psychiatry. We will then show how a framework inspired by reliability engineering can help understand the cognitive and affective dysfunctions of psychosis.

The neo-Kreapelinian framework is named for the founder of modern psychiatry (Kraepelin and Diefendorf, 1907), built on schemes such as the Feigner criteria (Feighner et al., 1972) and the Research Diagnostic Criteria (RDC; Spitzer et al., 1975), and was eventually codified in the 3rd edition of the DSM (DSM-III). These codes used symptoms to determine whether someone fulfilled the necessary and sufficient conditions for a diagnosis, which assumed the status of a natural category. Although this made it possible for patients to share a diagnosis without sharing any symptoms, this level of within-category variation was acceptable if the criteria increased diagnostic reliability and harmonized practice (but see Markon et al., 2011). Within this framework theories about how the neural functions of schizophrenia patients are distinct from the neural functions of depressed or bipolar patients were immediately salient and substantive. Four decades later, genetic, cellular, neural, cognitive and affective dysfunctions are known to be shared across many distinct diagnostic categories, and even an *optimal* neo-Kreapelinian scheme could not sort things out. Fifty percent of people diagnosed with schizophrenia will fulfill criteria for comorbid substance abuse, and 50% will fulfill criteria for depression (Buckley et al., 2009; for additional critique, see MacDonald, 2013). Such comorbidity likely derives from a shared vulnerability: the genetic correlation for common SNP’s between schizophrenia and bipolar disorder may be as high as 0.68, and between schizophrenia and depression may be as high as 0.47 (Lee et al., 2013). At this point the neo-Kraepelinian framework helps insurance adjusters more than researchers, clinicians or patients. For these reasons, there is momentum toward another framework that we will call informal reverse-engineering.

Informal reverse-engineering, already implicit in much psychopathological research, is codified in NIMH’s Research Domain Criteria (RDoC) program: “The mandate for RDoC is to consider psychopathology in terms of maladaptive extremes along a continuum of normal functioning, to promote a translational emphasis” (Ford et al., 2014, p. S296). The framework of RDoC is a matrix: rows are different functions from five categories (positive valence systems, negative valence systems, cognition, social processes and arousal); columns are levels of analysis ranging from genes to behavior and symptoms (Insel and Cuthbert, 2009; Sanislow et al., 2010; Cuthbert and Kozak, 2013; Ford et al., 2014). Patients can therefore be characterized in terms of many different functions and how those functions relate to biological processes or broader symptoms and social dysfunctions. Moving the focus toward functional deficits and away from the clinical constellations of the DSM may enable a tighter link between biology and behavior in neuropsychiatric research. However, a number of concerns have already arisen. First, by its structure, RDoC implies independance across the brains’ multitude of functions (rows in the RDoC matrix). Just as DSM categories are comorbid, so different RDoC functions are often correlated (reminiscent of general cognitive ability; see also Fowler et al., 2012). The authors of RDoC are aware of this, but there is little allowance in the framework for elaborating these relationships. Second, the RDoC posits that variation in these functions improves our ability to identify biological mechanisms more than clinical symptoms can. However, such functions may turn out to be as complicated as symptoms or diagnoses (Flint and Munafò, 2007; Kendler and Neale, 2010). Third, the relationship between these functions and symptoms is weak at best (Gold et al., 2012), and at times functions associated with very different neural structures correlate equally well with symptoms (MacDonald, 2013). The key shortcoming of the informal reverse-engineering approach in RDoC derives from isolating constructs from each other and thereby drawing attention away from the *structure* of psychopathology. Even when statistical relationships are discerned, it is not easy to append these into the cumulative science of mental illness.

This article will argue for a new framework, borrowed from reliability engineering, to guide the accumulation of knowledge and the development of new treatments for mental disorders. Our focus will be on psychosis, but many of these observations apply broadly to psychopathology. We will argue that adjusting the framework from which we motivate and report our findings is not only desirable, but is also a necessary step in the cognitive and affective neuroscience of these disorders. This new perspective based on a tool called *fault tree analysis* makes affordances for examining: (1) how causes that are neither necessary nor sufficient in and of themselves can result in psychosis; and how (2) within-diagnosis heterogeneity; and (3) co-morbidity arise.

## Fault Tree Analysis

**Figure 1:**
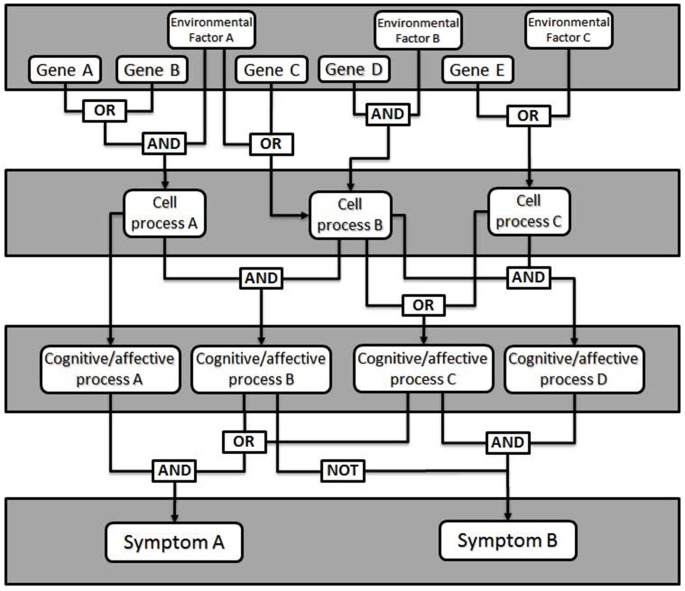
**General model of a fault tree.** Gene expression changes combine with environmental factors (e.g., stress, brain injury) to affect cellular level processes (e.g., synaptic plasticity, neurotransmitter release). Logical combinations of alterations in cellular level processes produce changes in particular cognitive or affective processes (e.g., working memory, mood), which manifest as clinical signs or symptoms. Additional logic combinations (e.g., critical mass: any 4 of 7, etc.) can also be modeled.

Fault tree analysis is a tool adapted from reliability engineering, in which the total likelihood of failure of a system is explained by the failure likelihood of each of its components (Rausand and Hoyland, 2004). Originally developed for analysis of rocket failures, fault trees seek to illustrate how faults of individual components interact within the system to cause an overall failure. To generate a fault tree, the different elements of the device must be identified, as well as their probability of failure—called faults—and how the faults interact and combine into failure modes (see Figure 1). A fault means the component is unable to perform its required function. Depending on other settings within the system, a small fault may be insignificant or trigger a cascade leading to a failure mode. In psychiatry, a failure mode is an impairment in some aspect of cognition, emotion or behavior of the kind we identify as a mental disorder.

The failure of a single component rarely results in a general system failure, due to built-in redundancy and plasticity of the brain. This enables most people with some forms of insult to function normally in the world. In this way, fault tree analysis makes affordances for causes that are neither necessary nor sufficient to cause dysfunction. Different individuals may come to a failure mode based on a different set of conditions, which contributes to within-diagnosis heterogeneity. Similarly, a given fault may lead to several failure modes, which gives rise to co-morbidity.

To generate a fault tree, one identifies the different components that contribute to the failure and for added value, quantify the likelihood of failure. Importantly, the fault tree illustrates how faults interact. This can be done with something like Boolean logic; faults may be related to other faults through AND or OR relationships (as well as others). This allows one to predict the probability of a failure mode given specific failures within the system. To this end, genetic mutations leading to psychiatric diseases may have measurable fault rates, although developmental and physiological failure rates will require more attention to estimate accurately. In addition, treatments can be modeled as externally-controlled variables in this network used to alter the outcome. The validity of the fault tree can then be tested by determining how well the known risks predict the known characteristics and rates of failure within a population.

Fault tree analysis is therefore a distinct and unused tool for understanding how different risk factors and therapies interact. Whereas the neo-Kraepelinian framework focuses on validating diagnoses and the informal reverse-engineering framework focuses on translational accounts of specific cognitive constructs, the reliability engineering framework attends to the ways in which faults combine into failure modes, and the ways in which the natural redundancies built into the brain can break down, or be built up. In this effort, computational models have a particularly important role.

### Computational Models as the Translational Glue within Fault Tree Analyses

In many respects computational models—mathematical formalizations of hypotheses—are already providing the kind of mechanistic understanding of mental disorders such as psychosis that fault tree analysis promises. Across all levels of analysis, computational models provide a means for describing how a system will respond to perturbations (for review, see Rolls et al., 2008). Such perturbations might be thought of as faults—specific malfunctions that either do or do not lead to a failure mode—or treatments intended to reverse those faults. Integrating computational modeling with fault-tree analysis in the context of psychiatric disease allows us to bridge from risk factors, whose rates serve as the parameters of the model, to disease pathophysiology, which form the modeled outcome. Such models suggest links between different faults, and how those faults at one scale of the fault tree lead to outcomes at another scale.

For example, genomic studies have identified many mutations related to schizophrenia, based on weak correlations in very large samples. These are not direct links from cause to behavioral outcome. More than 100 risk genes have been identified by GWAS linkage studies (Schizophrenia Working Group of the Psychiatric Genomics Consortium, 2014), and the final number of relevant mutations may be thousands. Computational models relate what we may know about the functions of these genes at the molecular and cellular scale to the observable changes in physiological biomarkers. The Hodgkin-Huxley neuronal models simulate ion channel and synaptic conductances to predict cellular dynamics. Channel mutations can be modeled by changing parameters and measuring the resulting changes in excitability and/or spiking patterns. Therefore, these models link changes at the protein scale to cellular scale biomarkers. At another scale, mean-field models simulate the average firing rates of populations of neurons in brain regions. These models can be used to relate changes in excitability or connection strengths to the emergence of synchrony and population oscillations that may be measured in a system-level biomarker, such as changes in functional MRI (fMRI), electroencephalogram (EEG) and even to cognitive deficits.

An advantage of computational models is that they can predict how interventions modulate neural activity to restore normal functioning. By testing these predictions from the models, we are inherently testing our underlying hypothesis of the physiological mechanisms of the disease state. Still, a challenge modelers share with experimentalists examining schizophrenia and other complex psychiatric disorders is how to integrate their models in a way that allows them to be placed within a larger, cumulative perspective of psychopathology. We provide the following example of how such findings can fit within a fault tree analysis.

### Generating a Fault Tree: An Example in Schizophrenia at the Cellular Level

**Figure 2:**
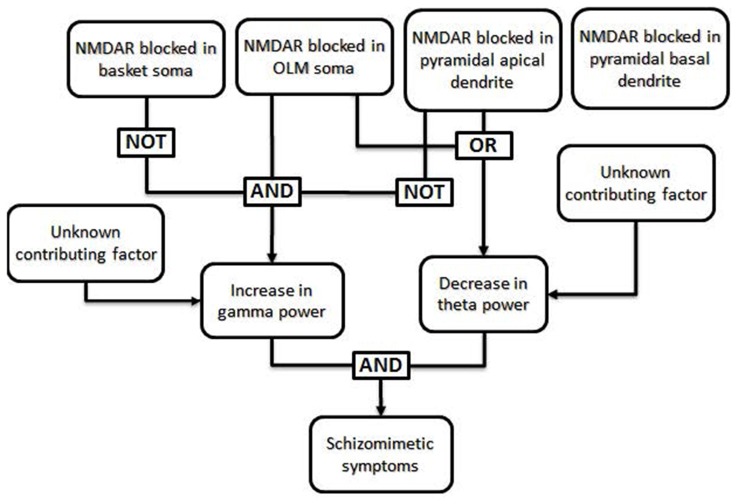
**Specific example of a fault tree generated from results of Neymotin et al. (2011).** The authors used a computational model to investigate the conditions under which the power of theta frequency oscillations (3–12 Hz) decrease while the power of gamma frequency oscillations (30–100 Hz) increase, as seen in animals and human patients after ketamine administration (Ehrlichman et al., 2009; Hong et al., 2010; Lazarewicz et al., 2010). In their computational model, a decrease in theta power resulted when NMDA receptors (NMDARs) were blocked in either the somas of oriens-lacunosum moleculare (OLM) cells or in the apical dendrites of pyramidal cells, regardless of the function of NMDARs in other cell types. In the same model, an increase in gamma power occurred only when NMDARs were blocked in the somas of OLM cells and NMDARs were *not* blocked in basket cells or the apical dendrites of pyramidal cells. Thus, the only combinations which generated both an increase in gamma power and a decrease in theta power involved blocking NMDARs in OLM soma with intact NMDARs in basket cells and pyramidal apical dendrites; the state of NMDARs in the pyramidal basal dendrites did not affect these results and can thus be said to be irrelevant in this case. Generation of a fault tree from these results allows one to visualize the roles that each factor plays in multiple downstream effects. Additional, unknown, factors may also impact the phenotype.

The work of Neymotin et al. (2011) can illustrate how a fault tree may be generated from the results of empirical research. The goal of this study was to simulate the effects of administration of the schizomimetic drug ketamine on hippocampal neuronal oscillations. Ketamine, an NMDA receptor (NMDAR) antagonist, elicits an increase in gamma (30–100 Hz) power and decrease in theta (3–12 Hz) power. The model consisted of 200 each of basket and oriens-lacunosum moleculare (OLM) interneurons and 800 hippocampal pyramidal cells and allowed them to test whether selectively blocking inhibitory circuits led to more high-frequency activity (Greene, 2001). NMDARs were present on the somas of each of the three cell types, as well as on the apical dendrites of pyramidal cells. Blocking NMDARs at all four locations decreased *both* theta and gamma power, which was inconsistent with ketamine effects in biological systems. A systematic investigation of all 16 possible combinations of insults demonstrated that pyramidal somatic NMDARs were largely irrelevant to theta and gamma power, whereas blocking pyramidal apical NMDARs alone was enough to decrease both theta and gamma power. The only condition which resulted in decreased theta and increased gamma was when OLM receptors were blocked while the basket interneurons and apical pyramidal receptors remained functional. This result can be translated into the Boolean logic illustrated in Figure 2.

The finding illustrates a helpful way of thinking: *how do different insults combine* to link NMDAR dysfunction to impairments in the cognitive and emotional functions of patients with schizophrenia? It may be that the cellular level of analysis is particularly amenable to fault tree analysis. Fault-tree models of cell-level malfunctions test causal relations between genetic mutations and changes in cell and network physiology, and then link these physiological changes to changes in cognitive and perceptual functions. Characterizing changes in brain function at the cellular level will require the development of appropriate animal models of psychiatric disease. Mutant mouse models link genetic mutations that increase risk for schizophrenia in humans to downstream changes in neural and network function. However, mouse models will be limited in the extent to which they can replicate cognitive and perceptual changes that occur in patients with schizophrenia, or the physiological dynamics of regions like the prefrontal cortex. Although it is difficult to relate cellular physiology and cortical network dynamics to downstream manifestations such as clinical symptoms, animal models can play an important role here. For example, ketamine administered to monkeys performing a cognitive control task show the specific pattern of errors that is a hallmark of cognitive control failure in patients with schizophrenia (Blackman et al., 2013). Neural recording during the period of synaptic malfunction also shows the underlying changes in cellular physiology and network oscillations that cause cognitive failures much like those seen in patients (Wang et al., 2013; Ma et al., 2015). Building links between synaptic, cell, and network phenomena in the context of behavior should serve to fill in key elements of fault tree networks.

There are other aspects of fault tree analysis that are not illustrated in Figure 2, for example the potential for two or more different combinations of insults to produce the same downstream effect, and modeling of non-binary outcomes using more complicated algorithms. A more complex model with more types of cells and insults may have revealed several mechanisms by which a decrease in theta power and increase in gamma power may result.

## A Fault Tree-Aligned Research Agenda: Implications for the Cognitive and Emotional Dysfunctions of Psychosis

It has been argued that schizophrenia is a syndrome not a disease: a set of symptoms, and other measurable signs, often seen together (Kotov et al., 2011; MacDonald, 2013). Assemblies of these failures, based on their relative co-occurrence, has led to our current diagnostic neo-Kraepelinian scheme. However, two patients labeled schizophrenic can both fulfill the current criteria without sharing any symptoms while simultaneously resembling patients not classified as schizophrenic. From a neo-Kraepelinian perspective, this is troubling. There is a clear need for a new framework that diagnoses patients in a new way.

Syndromal diseases are caused by multiple upstream faults resulting in a spectrum of symptoms, and no single cause defines the disease. The fault tree maps the likelihood of symptoms to the likelihood of cognitive and affective failures and in turn linking those failures to the likelihood of upstream faults in neural systems and genetic polymorphisms. This perspective allows for the overlap of symptoms seen across syndromes. The fault tree’s logic gates relate the different components of the genomics and physiology into a biometric. While the goal is to generate an overall model that explains the direct mechanistic connections underlying the indirect correlations, this can be approached one step at a time. Parts of a fault tree can be assembled from existing data, perhaps using probabilistic graphical models, to link different elements at adjacent scales and correlational relationships between elements at nonadjacent scales. For example, linking genetic mutations to cell pathophysiology, or cell excitability to network oscillations. But links also occur between scales that are not directly coupled, such as correlations between genes and disease prevalence. The fault tree can bring these lines of evidence together to make predictions for interventions that can be tested with computational models and validated in animal models and clinical studies. In this regard, studies of the neural systems of the cognitive and emotional dysfunctions in people with psychosis, including schizophrenia, will play a critical role.

There are limitations to the fault tree metaphor. Physiology may not fall into a neat tree-like structure. A tree implies that there is directionality in the causality: genes are responsible for cellular physiology and physiology is responsible for behavior. However, in biological systems there is clearly feedback between each of these levels, which will require attention. Furthermore, the schema presented here relies exclusively on dichotomous inputs and outputs. For the most part variables in neuroscience are measurable quantities, frequencies or probabilities. This concern has been considered and has been addressed within the field of reliability engineering. The expansion of the Boolean fault tree into one with more realistic kinds of variables is an additional refinement. Studies of patients can aid in this effort by providing more data about the distributions of their measured variables. For example, the relative skew of patient and control distributions for neural variables is relatively unknown; such data provide important additional information for validly predicting the likelihood of rare events. Another challenge is that fault tree analyses are top-down, and therefore, even with computational modeling, require a good deal of prior knowledge. One way to address this challenge in large data sets with insufficient prior knowledge is the use of probabilistic graphical models, which can bootstrap these efforts from the bottom-up.

The ultimate goal in developing a new diagnostic schema is to identify combination therapies that will maximize benefits. Fault trees can help identify how genetic, pharmacologic or behavioral therapies can improve, and inform cost/benefit analyses when considering treatment options. This approach may also inform public health policy, helping to identify disease mechanisms for which intervention has not yet been fully exploited. A fault tree provides a formal way of relating disparate sources of information about disease mechanisms and symptoms into one framework. Most importantly, it provides a path to move beyond a diagnostic scheme to one that directs therapies and research to maximize patient benefit.

## Funding

This work was supported in part by NIMH grant R01MH084861 (PI: AM3) and NSF career award 1135581 (PI: TN).

## Conflict of Interest Statement

The authors declare that the research was conducted in the absence of any commercial or financial relationships that could be construed as a potential conflict of interest.
